# Isolated Suprasellar Chordoma Mimicking Pituitary Adenoma: A Case Report

**DOI:** 10.7759/cureus.80647

**Published:** 2025-03-16

**Authors:** Mohammed K Al-Quwayee, Fahad Albadr, Rayan N Almasoud, Nawaf Alghamdi, Zeyad Abdulrahman Alhathloul, Mohamed Alquhidan

**Affiliations:** 1 Neuroradiology, King Saud University Medical City, Riyadh, SAU; 2 Radiology and Medical Imaging, King Saud University Medical City, Riyadh, SAU; 3 College of Medicine, King Saud University, Riyadh, SAU; 4 Internal Medicine, College of Medicine, King Saud bin Abdulaziz University for Health Sciences, Riyadh, SAU

**Keywords:** chordoma, mri, neoplasm, pituitary adenoma, suprasellar mass

## Abstract

Chordomas are uncommon neoplasms predominantly observed in adult males, typically middle-aged. These tumors present with diverse clinical features, including headaches, cranial neuropathies, and cerebrospinal fluid leakage. Surgical intervention is often complex due to tumor location or postresection reconstruction requirements, necessitating a multidisciplinary approach for optimal management. The report describes an atypical case of a 20-year-old female patient presenting with persistent headaches and visual disturbances. Magnetic resonance imaging (MRI) revealed a large sellar/suprasellar mass initially suggestive of a pituitary adenoma. Following surgical excision, histopathological analysis confirmed a chordoma. This report emphasizes the diagnostic and therapeutic challenges of suprasellar chordomas, which may radiologically mimic common sellar lesions. The case underscores the importance of including chordoma in the differential diagnosis of sellar/suprasellar masses, especially in younger patients with visual impairment and headache.

## Introduction

Chordoma, a rare malignant neoplasm arising from embryonic notochord remnants, predominantly occurs in the axial skeleton, including the skull base, spine, and sacrococcygeal region. Despite being histopathologically classified as low to intermediate grade, this tumor exhibits locally aggressive growth and destructive potential [1]. Chordomas occur at an incidence of 0.8 per million individuals annually [2]. Anatomically, approximately 50% arise in the sacrococcygeal region, 35% in the skull base, and 15% in the mobile spine [3]. These tumors exhibit a male predominance and are diagnosed more frequently in adults than in pediatric populations [2-4]. Clinically, patients often present with diplopia and headaches [5,6], with abducens nerve palsy being the most common neurological deficit [6]. Diagnostic ambiguity arises due to its radiological similarity to pituitary adenoma on cross-sectional imaging, often leading to misinterpretation. We present a 20-year-old female patient with a chordoma radiologically mimicking a pituitary adenoma.

## Case presentation

A 20-year-old female patient presented with double-vision, left-sided lateral peripheral visual loss, and occasional blurry vision associated with intermittent headaches for four months. On initial physical examination, the pupils were equally reactive to light with a normal red reflex. Limited abduction of the left eye and horizontal binocular diplopia were observed. Other cranial nerve examinations were normal, with no additional neurological deficits.

**Figure 1:**
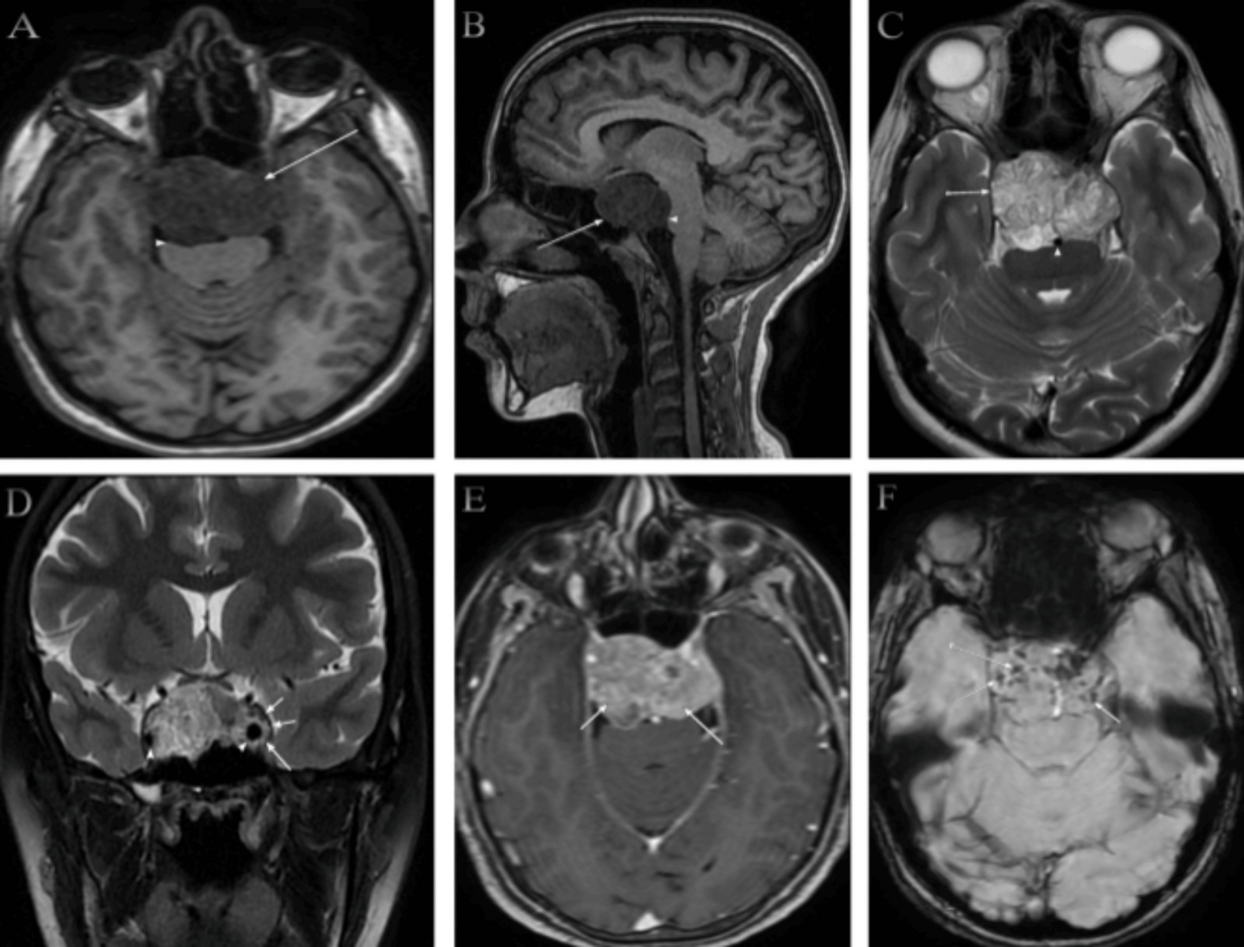
Preoperative brain magnetic resonance imaging (MRI) T1-weighted images on axial (A) demonstrate predominantly intermediate signal intensity (long arrow) with significant mass effect on the brain stem (arrowhead). T1-weighted images on sagittal (B) also demonstrate predominantly intermediate signal intensity (long arrow) with significant mass effect on the brain stem (arrowhead). T2-weighted images on axial (C) demonstrate heterogeneous hyperintensity (long arrow) with partial encasement of the basilar artery without vascular narrowing (arrowhead). T2-weighted images on the coronal plane (D) show bilateral invasion of the cavernous sinuses, more pronounced on the left side (long arrow), with encasement of the internal carotid arteries bilaterally (arrowhead), without vascular narrowing. Contrast-enhanced fat-suppressed T1-weighted images on the axial plane (E) show moderate heterogeneous enhancement. Susceptibility-weighted imaging (SWI) on the axial plane (F) shows foci of susceptibility effect involving the periphery of the mass, likely representing calcification

Preoperative brain magnetic resonance imaging (MRI) revealed a large sellar/suprasellar mass measuring approximately 38.5 x 46.2 x 25.2 mm in the anteroposterior, transverse, and craniocaudal dimensions, respectively, with predominantly intermediate signal intensity with significant mass effect on the brain stem (Figures 1A, 1B). The T2-weighted images demonstrate heterogeneous hyperintensity with partial encasement of the basilar artery, without evidence of vascular narrowing (Figure 1C). Additionally, the images reveal bilateral invasion of the cavernous sinuses, more prominent on the left side, with encasement of the internal carotid arteries bilaterally, without vascular narrowing (Figure 1D). Contrast-enhanced, fat-suppressed T1-weighted imaging illustrates moderate heterogeneous enhancement (Figure 1E). Susceptibility-weighted imaging (SWI) reveals foci of susceptibility effects involving the periphery of the mass, likely indicative of calcification (Figure 1F).

**Figure 2:**
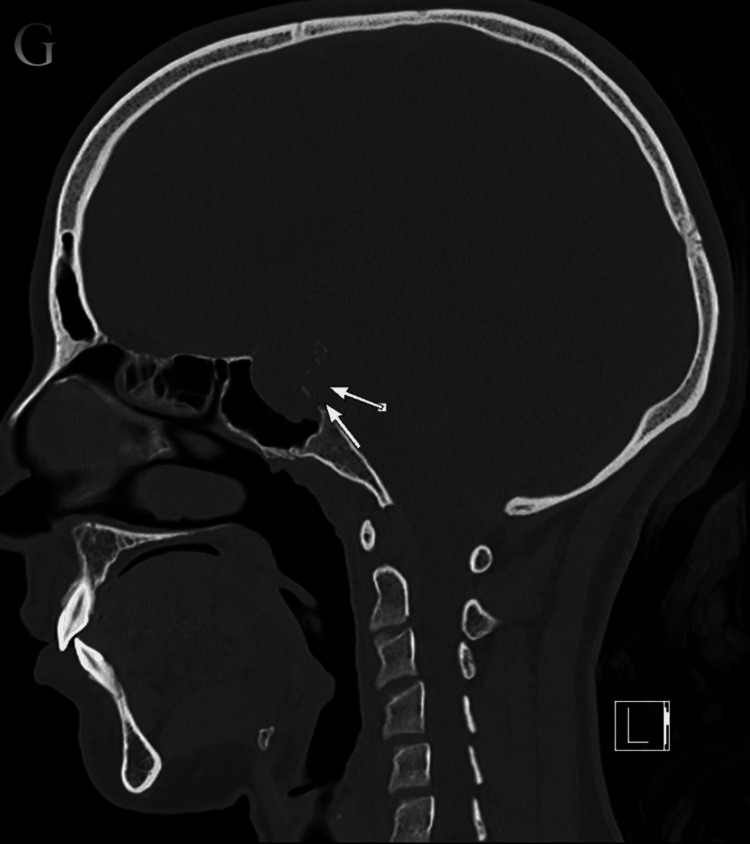
In the brain CT on sagittal plane bone window (G), the lesion was causing destruction (long arrows) of the pituitary fossa, dorsum sellae, and tuberculum CT: computed tomography

**Figure 3:**
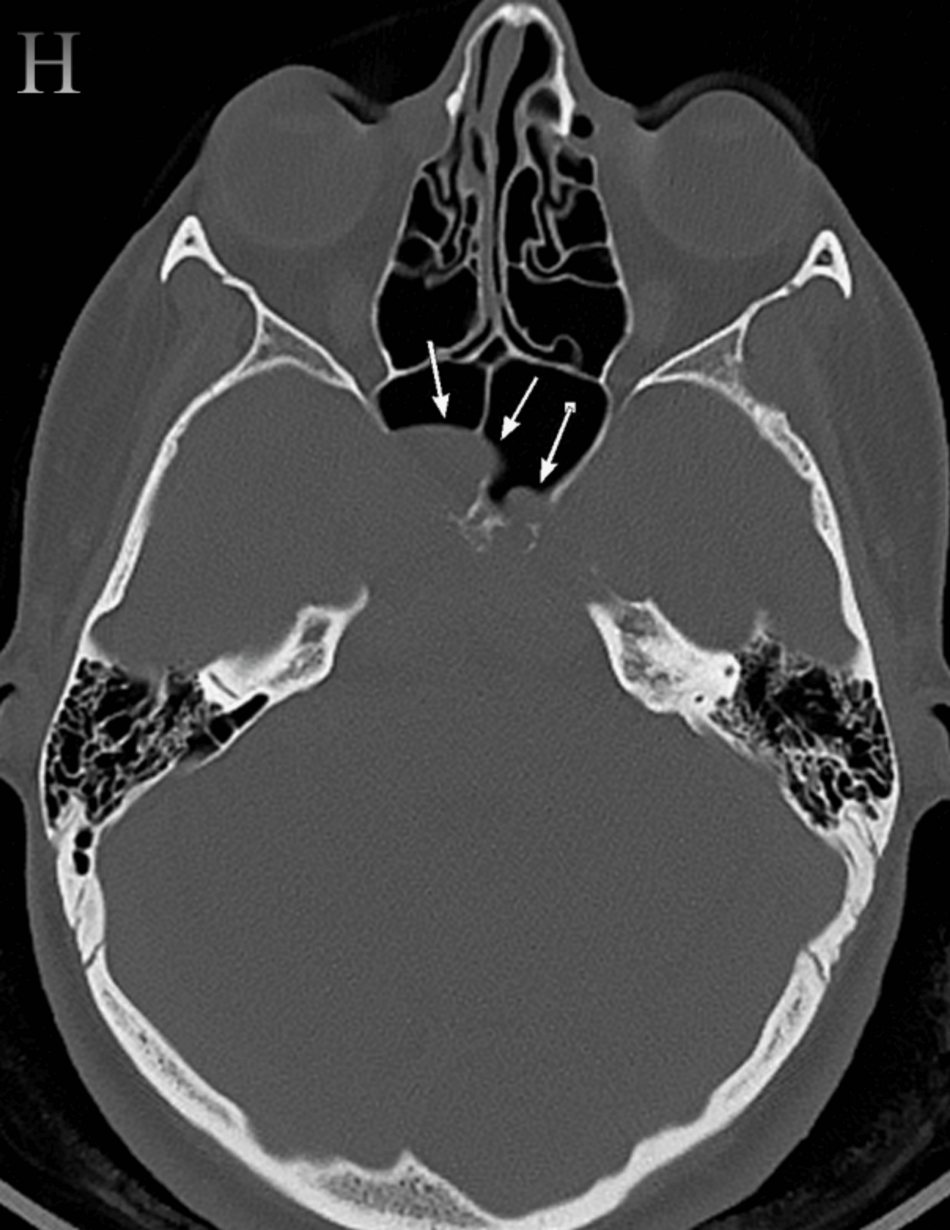
Bone window axial CT (H) shows extension into the sphenoid sinus (long arrows) CT: computed tomography

**Figure 4:**
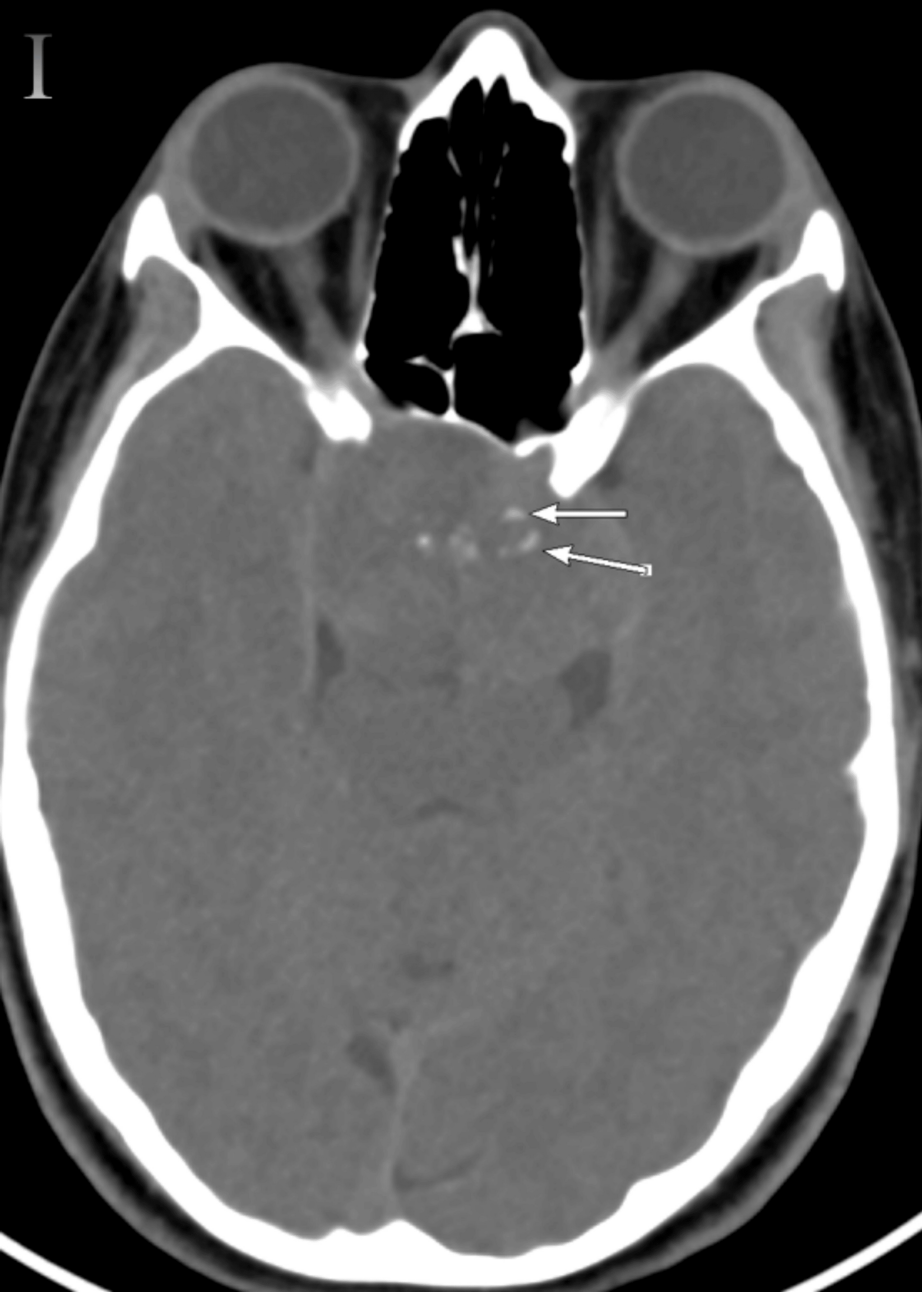
Soft window axial CT (I) shows several flecks of calcification (long arrows) CT: computed tomography

The brain computed tomography (CT) on the sagittal plane bone window revealed a lesion causing destruction of the pituitary fossa, dorsum sellae, and tuberculum (Figure 2), with further extension into the sphenoid sinus (Figure 3), and several flecks of calcification observed on the soft window CT (Figure 4).

Surgical resection of the tumor was performed using an endoscopic transnasal approach. Upon opening the sellar floor, the tumor appeared fibrous and lobulated. Intraoperative frozen biopsy and immunohistochemical staining confirmed the diagnosis of chordoma. Subtotal resection was achieved without complications, and postoperative MRI confirmed near-complete tumor removal. Fortunately, in our case, the patient did not experience postoperative complications, and all symptoms resolved completely. The patient was subsequently referred to the radiation oncology department for further treatment discussions. Given the histopathological diagnosis, high-dose proton therapy is the standard of care for chordoma. However, due to the unavailability of proton therapy at our institution, the patient sought treatment abroad and completed a proton therapy regimen in Germany.

## Discussion

The current report presents a case of a 20-year-old female patient with a suprasellar chordoma radiologically mimicking a pituitary adenoma. Chordomas are low-grade, slow-growing, yet locally aggressive sarcomas originating from notochord remnants. These tumors typically arise along the midline axial skeleton, spanning the clivus to the sacrococcygeal region. Representing approximately 3% of primary bone malignancies [7], their indolent growth often delays diagnosis, with symptoms evolving insidiously over months to years, depending on tumor location and extent [8]. Clinical presentation correlates with anatomical site, commonly featuring localized pain or neurological deficits [8]. This report highlights a suprasellar chordoma in a young female, an atypical demographic for this tumor. Key imaging and histopathological findings align with prior literature [5]. 

While clinical suspicion initiates evaluation, cross-sectional imaging (MRI/CT) remains critical for accurate diagnosis, preoperative planning, and biopsy guidance [9]. Multidisciplinary collaboration ensures optimal management of these complex lesions.

Chordomas and pituitary adenomas are clinically distinct tumors with unique radiological features. Chordomas originate from notochord remnants and predominantly arise along the axial skeleton (sacrum, clivus, and vertebrae). CT imaging reveals lytic bone destruction with intratumoral calcifications and cortical erosion [10]. MRI typically shows lobulated masses with heterogeneous T2 hyperintensity (reflecting mucoid or cystic components) and irregular contrast enhancement, often compressing posterior structures such as the brainstem [11].

Pituitary adenomas originate within the sella turcica. CT may detect sellar enlargement, floor erosion, or sphenoid sinus invasion, but calcifications are rare [12]. MRI usually depicts a well-circumscribed sellar mass with homogeneous contrast enhancement, though signal intensity varies on T1/T2 sequences depending on secretory activity (e.g., T1 hyperintensity in prolactinomas) [13]. 

Compared to CT, MRI has limitations in evaluating calcifications and accurately assessing skull base osteolysis, particularly in the skull base foramina. CT is necessary to determine the extent of bone involvement and detect calcification patterns within the lesion [14]. In many cases, a needle or open biopsy is performed to confirm the diagnosis [7].

Chordomas are aggressive malignancies with variable behavior, and some patients may experience rapid progression [3]. The primary treatment is en bloc resection; however, the axial location often makes complete resection challenging. When surgical removal is not feasible, radiation therapy is typically administered, with adjuvant radiation considered in certain cases [2]. 

## Conclusions

Chordoma, a malignant neoplasm originating from notochord remnants, typically develops along the midline of the spinal axis. While most cases involve the sacrum, skull base, or vertebrae, suprasellar presentations are exceedingly rare and predominantly affect middle-aged males. This report highlights an unusual occurrence in a 20-year-old female patient. Effective management of chordoma requires collaboration among a multidisciplinary team, including otolaryngologists, neurosurgeons, pathologists, and radiologists, to optimize diagnostic accuracy and therapeutic outcomes.

## References

[1] Barber SM, Sadrameli SS, Lee JJ (2021). Chordoma-current understanding and modern treatment paradigms. J Clin Med.

[2] Ulici V, Hart J (2022). Chordoma: a review and differential diagnosis. Arch Pathol Lab Med.

[3] Wedekind MF, Widemann BC, Cote G (2021). Chordoma: current status, problems, and future directions. Curr Probl Cancer.

[4] Udoh MO, Imasogie DE, Udoh DO (2021). Skull base chordoma: a case presentation and review of literature. West Afr J Med.

[5] Shin JH, Cho DS, Kim MH, Kim SH (2004). Sellar chordoma mimicking pituitary adenoma. J Korean Neurosurg Soc.

[6] Favre J, Deruaz JP, Uske A, de Tribolet N (1994). Skull base chordomas: presentation of six cases and review of the literature. J Clin Neurosc.

[7] Tenny S, Varacallo MA (2025). Chordoma. https://pubmed.ncbi.nlm.nih.gov/28613596/.

[8] Karele EN, Paze AN (2022). Chordoma: to know means to recognize. Biochim Biophys Acta Rev Cancer.

[9] Reeves RA, Parekh M (2025). Pituitary gland imaging. https://pubmed.ncbi.nlm.nih.gov/32310449/.

[10] Erdem E, Angtuaco EC, Van Hemert R, Park JS, Al-Mefty O (2003). Comprehensive review of intracranial chordoma. Radiographics.

[11] Doucet V, Peretti-Viton P, Figarella-Branger D, Manera L, Salamon G (1997). MRI of intracranial chordomas. Extent of tumour and contrast enhancement: criteria for differential diagnosis. Neuroradiology.

[12] Davis PC, Hoffman JC Jr, Spencer T, Tindall GT, Braun IF (1987). MR imaging of pituitary adenoma: CT, clinical, and surgical correlation. AJR Am J Roentgenol.

[13] Pisaneschi M, Kapoor G (2005). Imaging the sella and parasellar region. Neuroimaging Clin N Am.

[14] Masino F, Montatore M, Balbino M, Andrea D'Arma GM, Muscatella G, Gifuni R, Guglielmi G (2024). Diagnosis and management of a rare case of clival chordoma in a young male patient. Radiol Case Rep.

